# Supplementary material for: Piperlongumine Analogs Promote A549 Cell Apoptosis through Enhancing ROS Generation
Source: Molecules. 2021 May 28;26(11):3243. doi: 10.3390/molecules26113243 (PMC8198376; doi:10.3390/molecules26113243)
Supplement: Supplementary file 1 [file molecules-26-03243-s001.zip › molecules-1201252-supplementary.pdf]

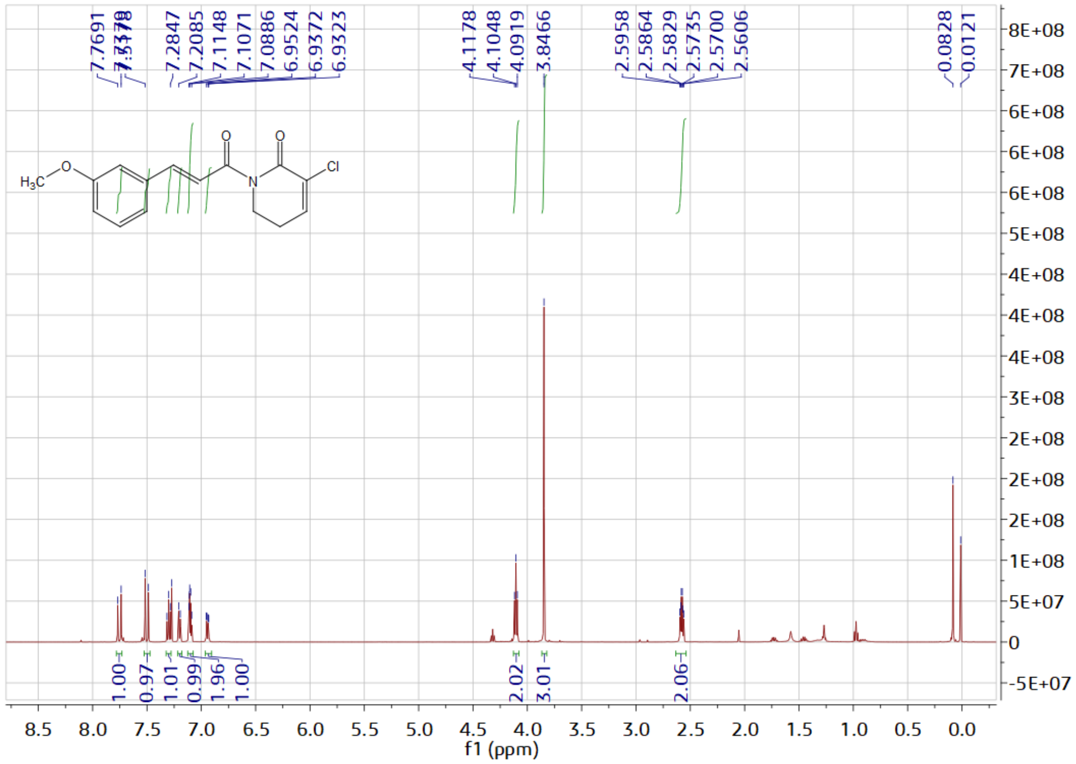

7.7691
7.7378
7.5178
7.2847
7.2085
7.1148
7.1071
7.0886
6.9524
6.9372
6.9323
4.1178
4.1048
4.0919
3.8466
2.5958
2.5864
2.5829
2.5735
2.5700
2.5606
0.0828
0.0121
1.00
0.97
1.01
0.99
1.96
1.00
2.02
3.01
2.06
f1 (ppm)

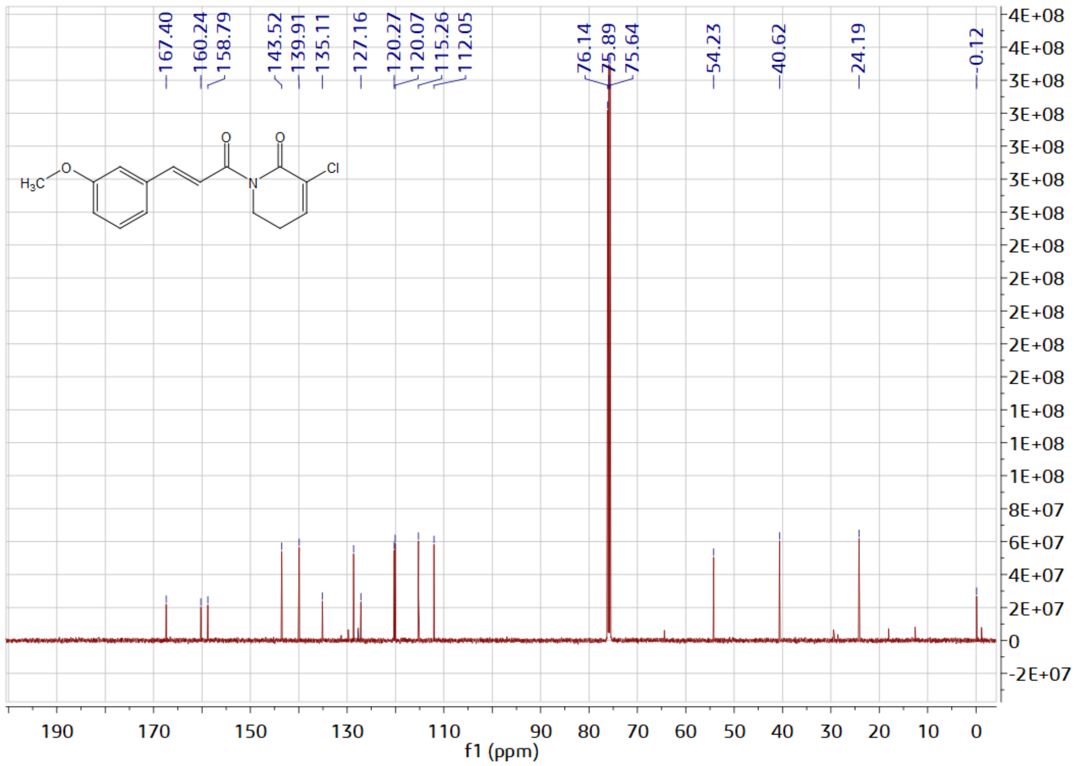

167.40
160.24
158.79
143.52
139.91
135.11
127.16
120.27
120.07
115.26
112.05
76.14
75.89
75.64
54.23
40.62
24.19
-0.12
f1 (ppm)

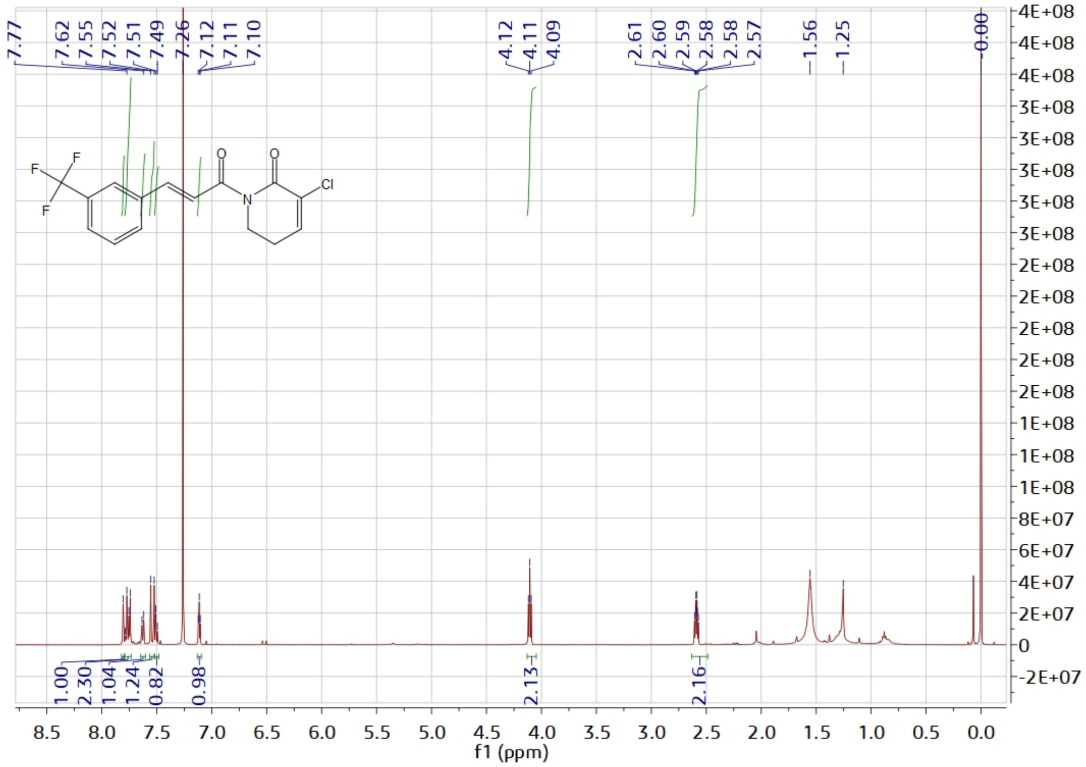
7.77
7.62
7.55
7.52
7.51
7.49
7.26
7.12
7.11
7.10
4.12
4.11
4.09
2.61
2.60
2.59
2.58
2.58
2.57
1.56
1.25
0.00
1.00
2.30
1.04
1.24
0.82
0.98
2.13
2.16
F
F
F
O
O
N
Cl
f1 (ppm)

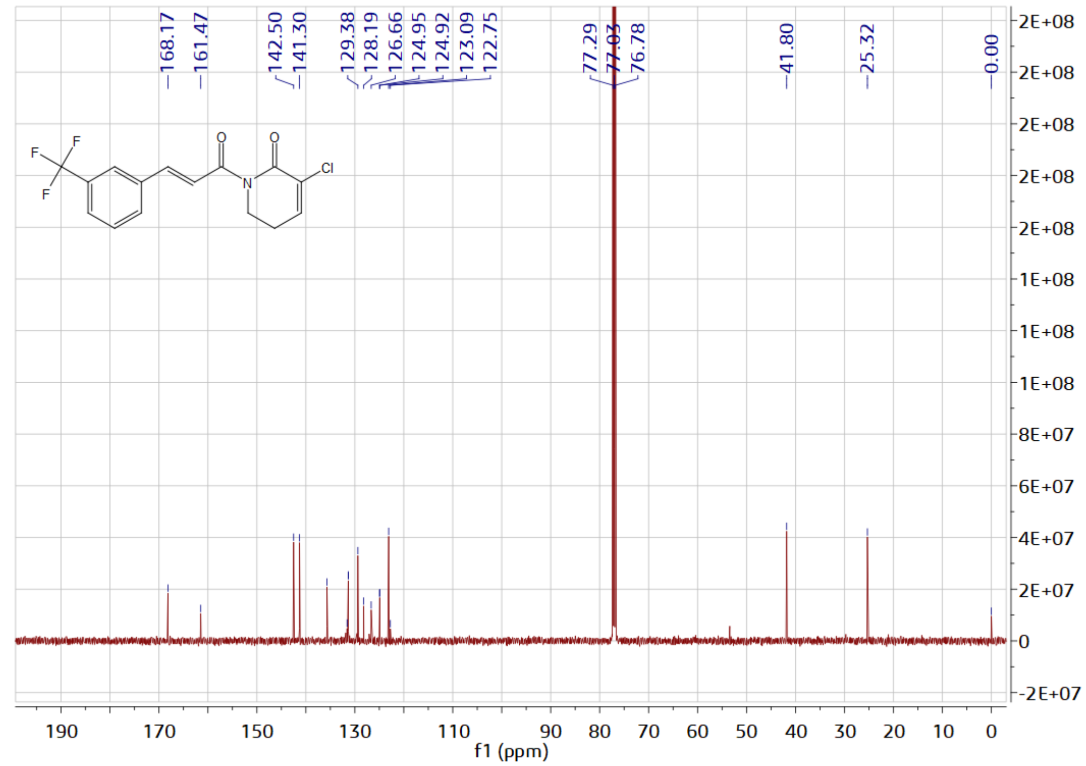
168.17
161.47
142.50
141.30
129.38
128.19
126.66
124.95
124.92
123.09
122.75
77.29
77.03
76.78
41.80
25.32
0.00
F
F
F
O
O
N
Cl
f1 (ppm)

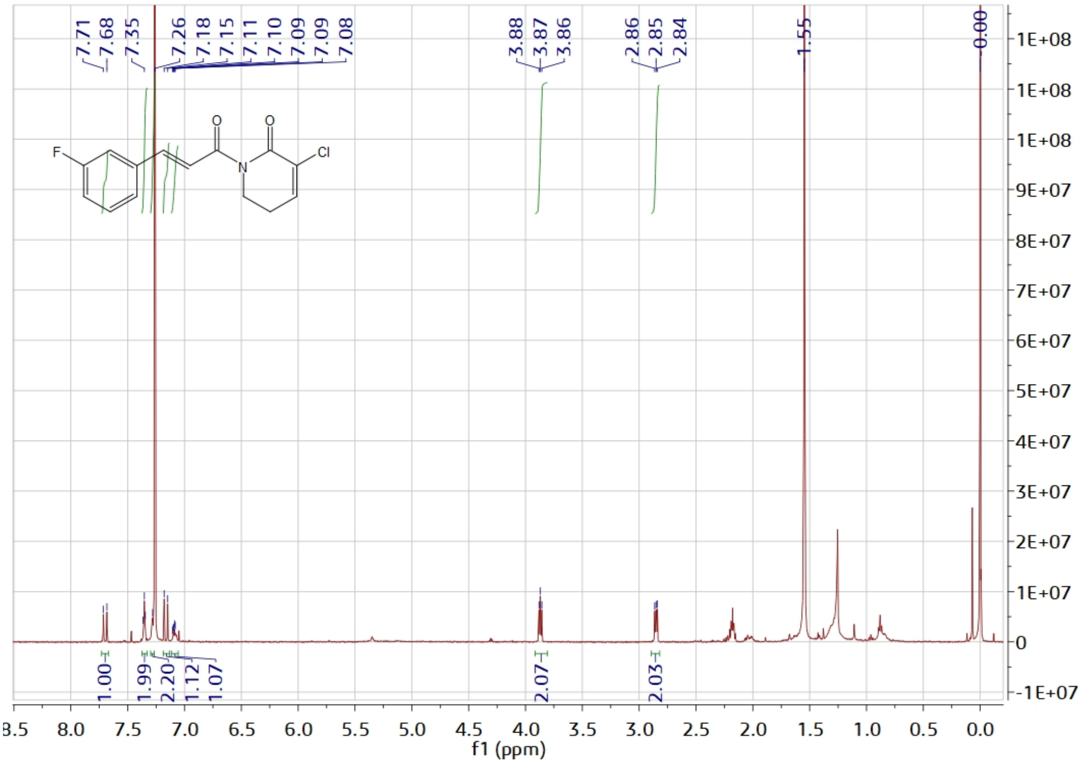
7.71
7.68
7.35
7.26
7.18
7.15
7.11
7.10
7.09
7.09
7.08
3.88
3.87
3.86
2.86
2.85
2.84
1.55
0.00
F
O
O
N
Cl
1.00
1.99
2.20
1.12
1.07
2.07
2.03
f1 (ppm)

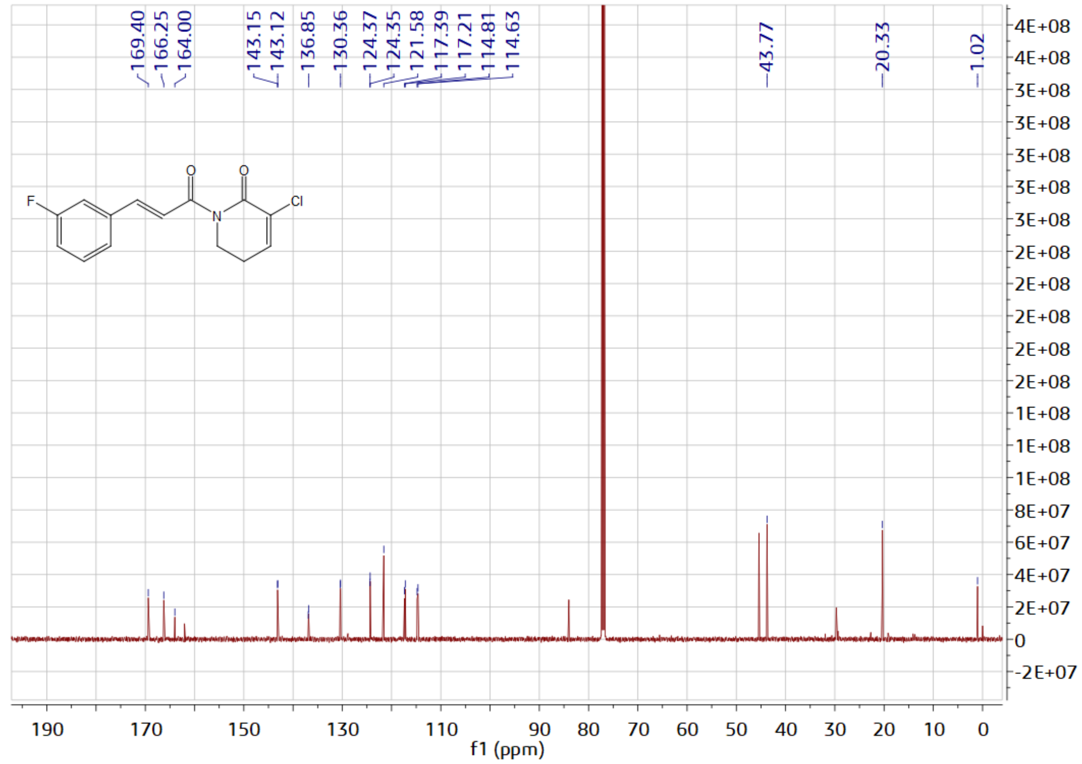
169.40
166.25
164.00
143.15
143.12
136.85
130.36
124.37
124.35
121.58
117.39
117.21
114.81
114.63
43.77
20.33
1.02
F
O
O
N
Cl
f1 (ppm)

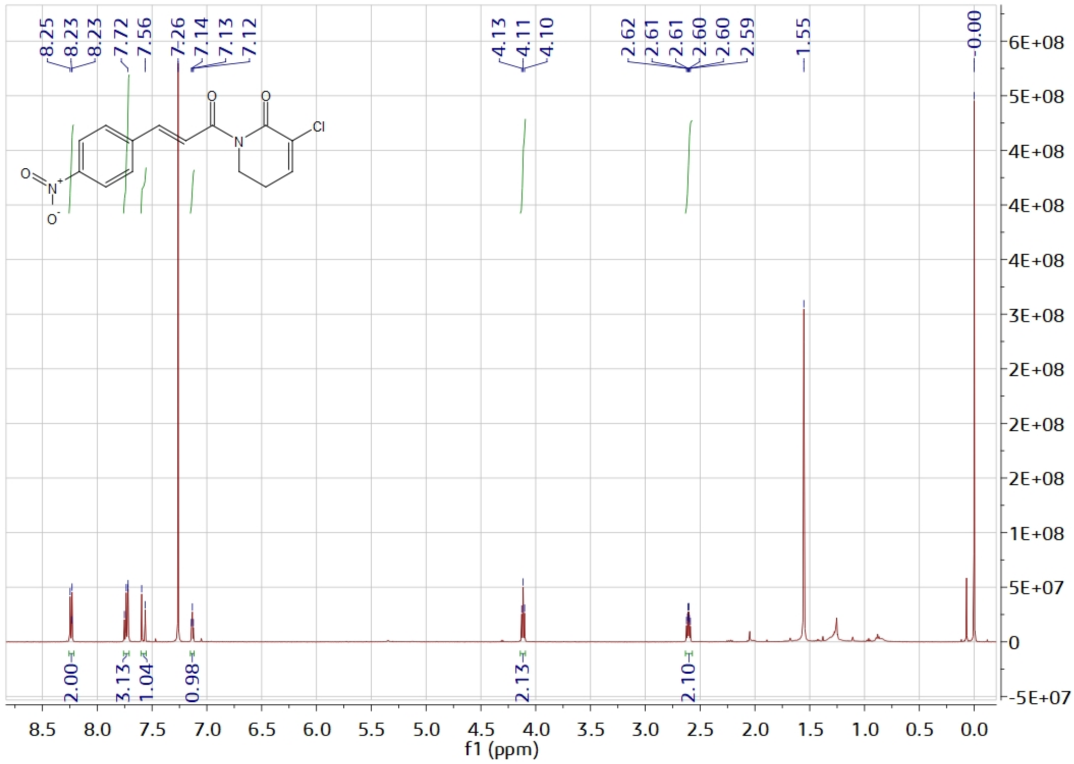
8.25
8.23
8.23
7.72
7.56
7.26
7.14
7.13
7.12
4.13
4.11
4.10
2.62
2.61
2.61
2.60
2.60
2.59
1.55
0.00
2.00
3.13
1.04
0.98
2.13
2.10
f1 (ppm)

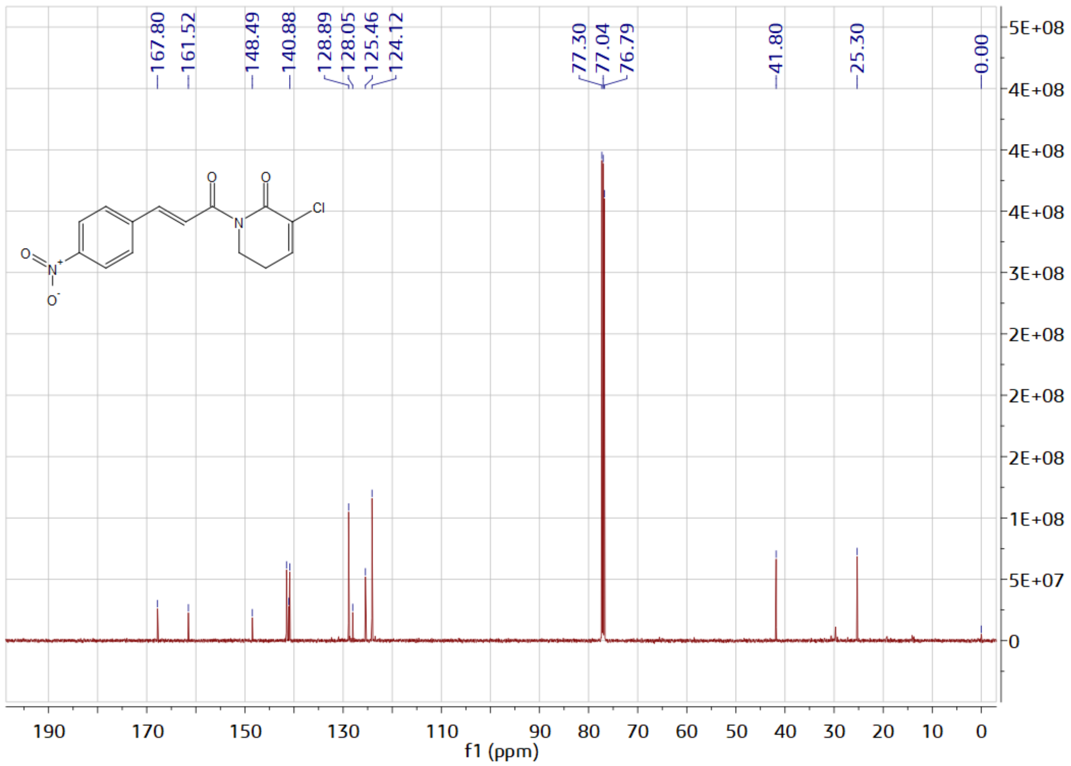
167.80
161.52
148.49
140.88
128.89
128.05
125.46
124.12
77.30
77.04
76.79
41.80
25.30
0.00
f1 (ppm)

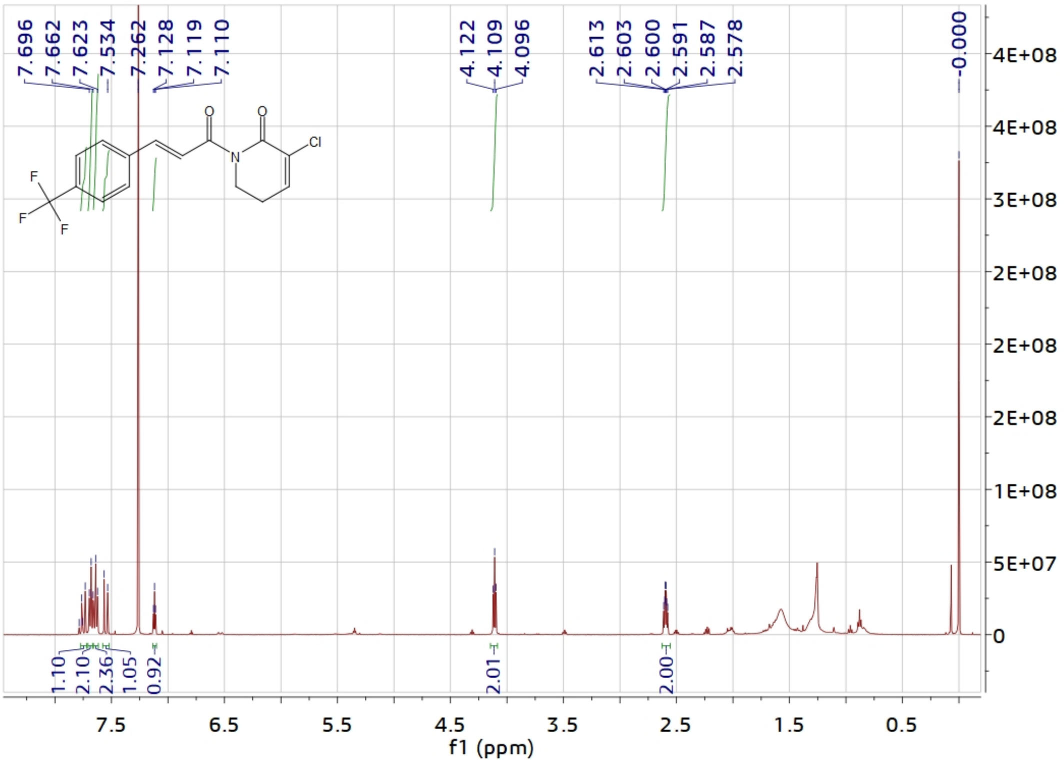
7.696
7.662
7.623
7.534
7.262
7.128
7.119
7.110
4.122
4.109
4.096
2.613
2.603
2.600
2.591
2.587
2.578
-0.000
F
F
F
O
O
N
Cl
1.10
2.10
2.36
1.05
0.92
2.01
2.00
4E+08
3E+08
2E+08
1E+08
5E+07
0
7.5
6.5
5.5
4.5
3.5
2.5
1.5
0.5
f1 (ppm)

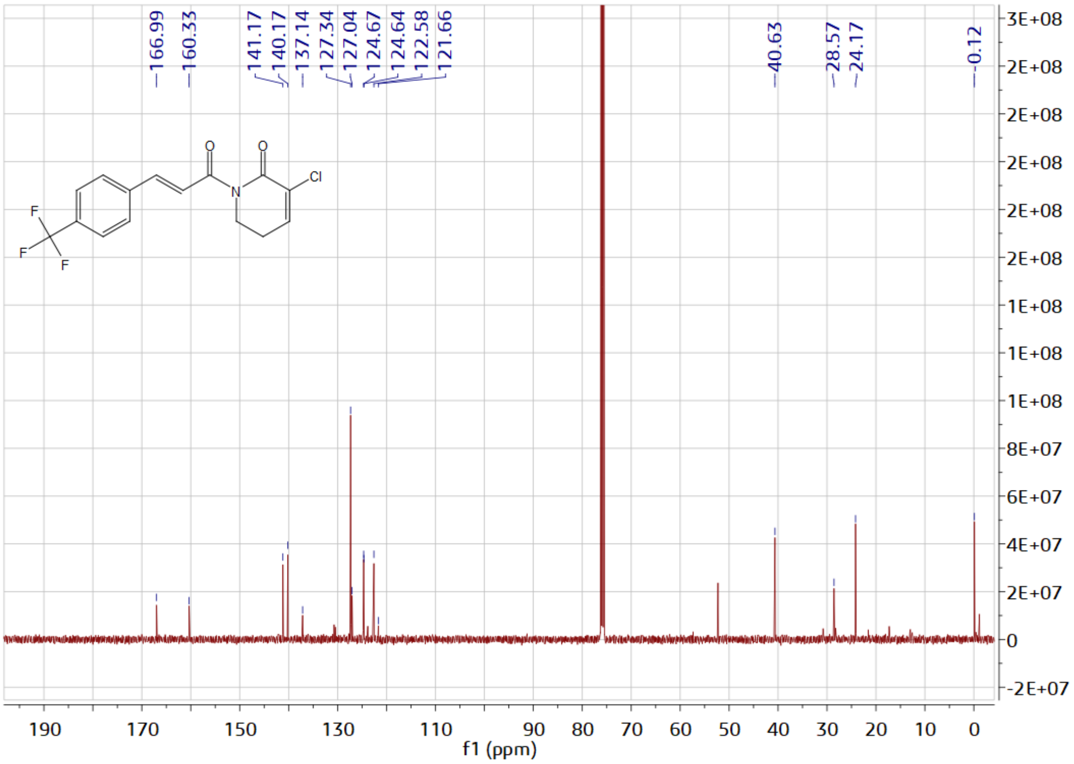
166.99
160.33
141.17
140.17
137.14
127.34
127.04
124.67
124.64
122.58
121.66
40.63
28.57
24.17
-0.12
F
F
F
O
O
N
Cl
3E+08
2E+08
1E+08
8E+07
6E+07
4E+07
2E+07
0
-2E+07
190
170
150
130
110
90
80
70
60
50
40
30
20
10
0
f1 (ppm)

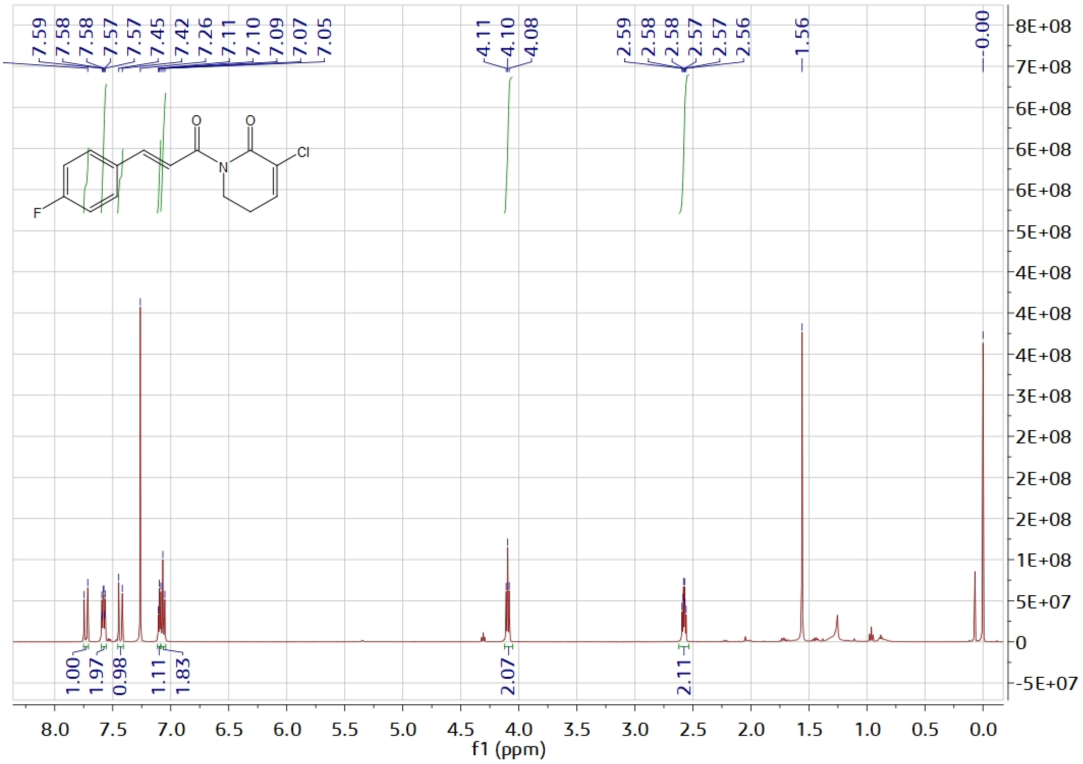
7.59
7.58
7.58
7.57
7.57
7.45
7.42
7.26
7.11
7.10
7.09
7.07
7.05
4.11
4.10
4.08
2.59
2.58
2.58
2.57
2.57
2.56
1.56
0.00
1.00
1.97
0.98
1.11
1.83
2.07
2.11
f1 (ppm)
F
O
O
N
Cl

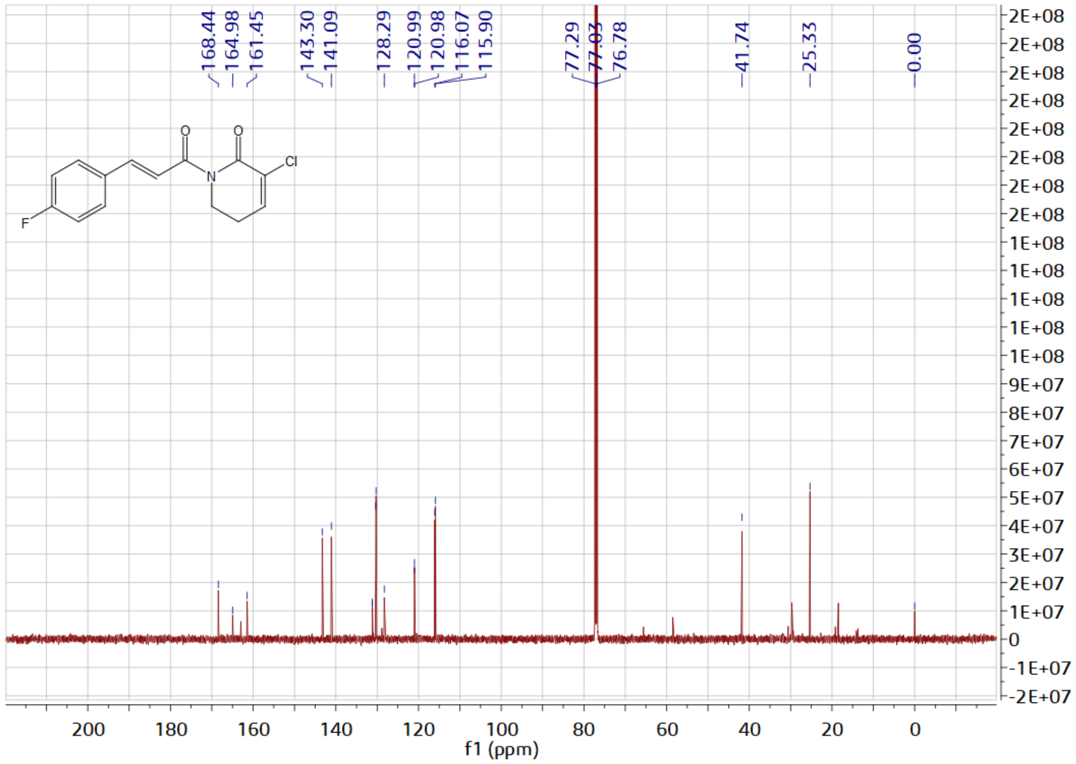
168.44
164.98
161.45
143.30
141.09
128.29
120.99
120.98
116.07
115.90
77.29
77.03
76.78
41.74
25.33
0.00
f1 (ppm)
F
O
O
N
Cl

PL-1

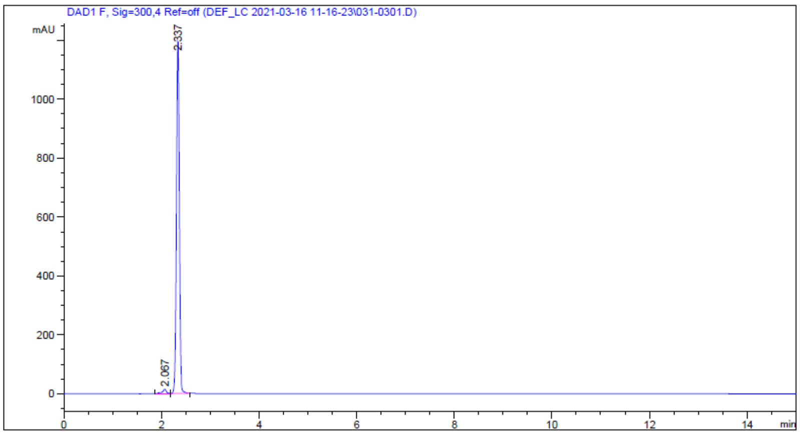

==
面积百分比报告
==

排序 : 信号
乘积因子: : 1.0000
稀释因子: : 1.0000
内标使用乘积因子和稀释因子

信号 1: DAD1 F, Sig=300,4 Ref=off

峰 #	保留时间 [min]	类型	峰宽 [min]	峰面积 [mAU*s]	峰高 [mAU]	峰面积 %
1	2.067	BV	0.0772	79.69137	14.70570	1.6151
2	2.337	VB	0.0631	4854.35156	1203.37988	98.3849

PL-2

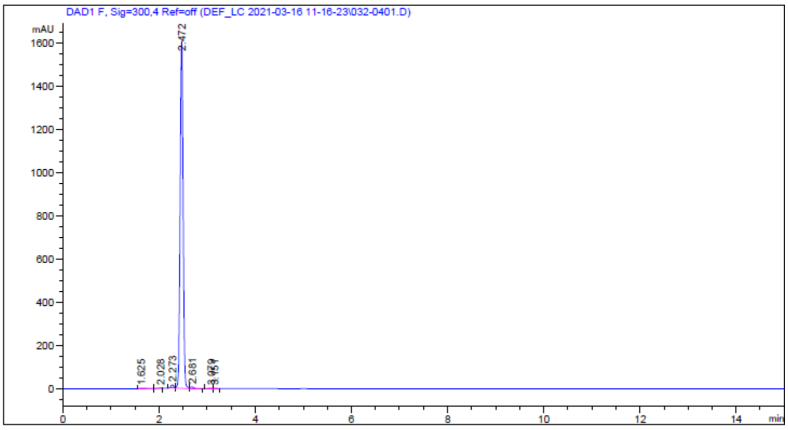

==
面积百分比报告
==

排序 : 信号
乘积因子: : 1.0000
稀释因子: : 1.0000
内标使用乘积因子和稀释因子

信号 1: DAD1 F, Sig=300,4 Ref=off

峰 #	保留时间 [min]	类型	峰宽 [min]	峰面积 [mAU*s]	峰高 [mAU]	峰面积 %
1	1.625	BV	0.1462	20.49888	1.95744	0.2953
2	2.028	VB	0.0591	6.32188	1.63441	0.0911
3	2.273	BV	0.0638	67.84969	15.90887	0.9774
4	2.472	VV	0.0651	6782.48633	1610.90503	97.6994
5	2.681	VB	0.0803	39.43160	7.15878	0.5680
6	3.079	BV	0.0757	12.71709	2.57003	0.1832
7	3.151	VB	0.0759	12.89151	2.59769	0.1857

PL-3

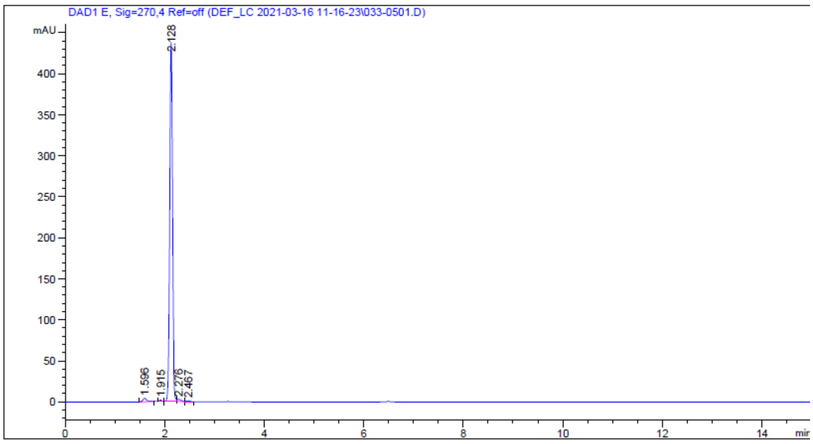

==
面积百分比报告
==

排序 : 信号
乘积因子: : 1.0000
稀释因子: : 1.0000
内标使用乘积因子和稀释因子

信号 1: DAD1 E, Sig=270,4 Ref=off

峰 #	保留时间 [min]	类型	峰宽 [min]	峰面积 [mAU*s]	峰高 [mAU]	峰面积 %
1	1.596	BB	0.0821	23.56453	4.16191	1.3377
2	1.915	BB	0.0509	5.59526	1.77025	0.3176
3	2.128	BV	0.0616	1712.84729	438.32520	97.2313
4	2.276	VV	0.0769	14.05293	2.69304	0.7977
5	2.467	VB	0.0739	5.56099	1.12106	0.3157

PL-4

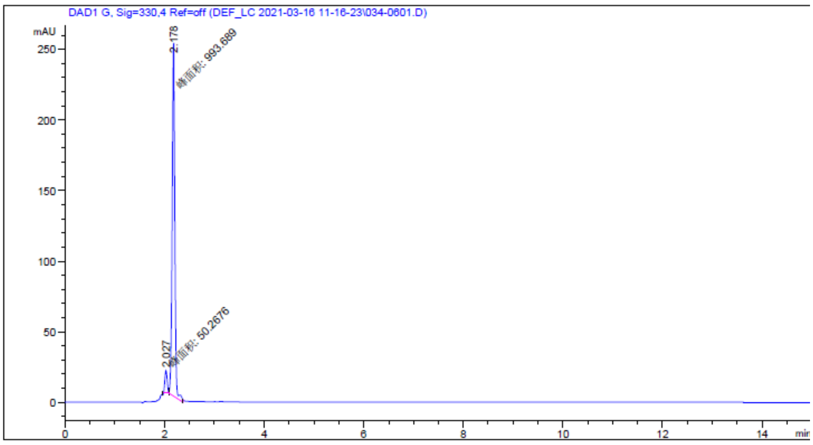

==
面积百分比报告
==

排序 : 信号
乘积因子: : 1.0000
稀释因子: : 1.0000
内标使用乘积因子和稀释因子

信号 1: DAD1 G, Sig=330,4 Ref=off

峰 #	保留时间 [min]	类型	峰宽 [min]	峰面积 [mAU*s]	峰高 [mAU]	峰面积 %
1	2.027	MM	0.0520	50.26759	16.09933	4.8151
2	2.178	MM	0.0657	993.68854	252.06909	95.1849

PL-5

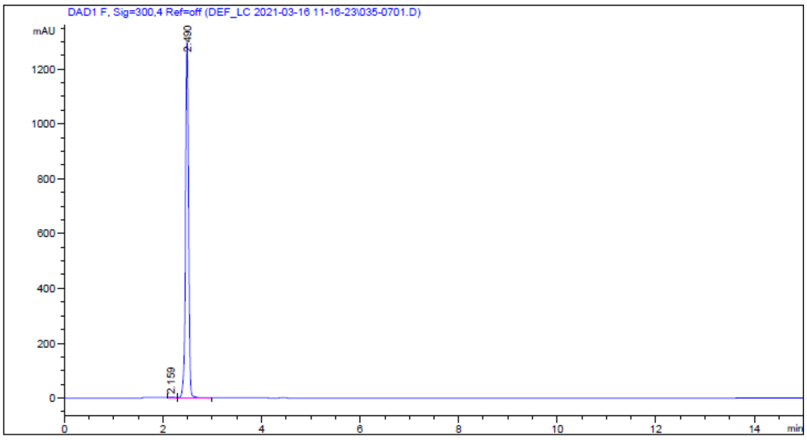

===

面积百分比报告

===

排序 : 信号
乘积因子: : 1.0000
稀释因子: : 1.0000
内标使用乘积因子和稀释因子

信号 1: DAD1 F, Sig=300,4 Ref=off

峰 #	保留时间 [min]	类型	峰宽 [min]	峰面积 [mAU*s]	峰高 [mAU]	峰面积 %
1	2.159	VV	0.1123	15.16134	1.76409	0.2649
2	2.490	VB	0.0670	5708.13770	1305.98132	99.7351

PL-6

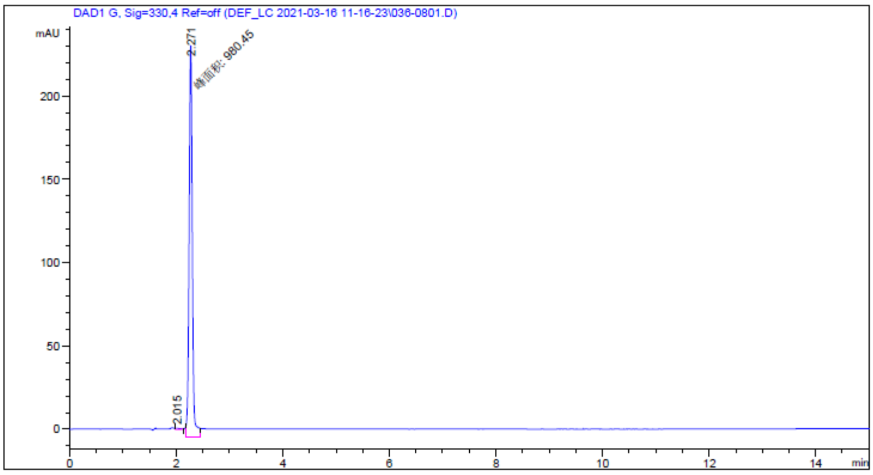

===

面积百分比报告

===

排序 : 信号
乘积因子: : 1.0000
稀释因子: : 1.0000
内标使用乘积因子和稀释因子

信号 1: DAD1 G, Sig=330,4 Ref=off

峰 #	保留时间 [min]	类型	峰宽 [min]	峰面积 [mAU*s]	峰高 [mAU]	峰面积 %
1	2.015	VV	0.0889	5.30532	8.04854e-1	0.5382
2	2.271	MM	0.0691	980.45038	236.46912	99.4618
